# Medication adherence in thoracic transplant candidates

**DOI:** 10.3389/fpsyg.2026.1726871

**Published:** 2026-03-20

**Authors:** David M. Sanborn, Kelly M. Pennington, Roberto P. Benzo, Terry D. Schneekloth, David B. Erasmus, Marie Budev, Satish Chandrashekaran, Erika D. Lease, Deborah J. Levine, Cassie L. Zell, Paul J. Novotny, Daniel S. Yip, Sudhir S. Kushwaha, Brian Hardaway, Cassie C. Kennedy

**Affiliations:** 1 Division of Pulmonary and Critical Care Medicine, Department of Medicine, Mayo Clinic, Rochester, MN, United States; 2 William J. Von Liebig Center for Transplantation and Clinical Regeneration, Mayo Clinic, Rochester, MN, United States; 3 Department of Psychiatry and Psychology, Mayo Clinic, Rochester, MN, United States; 4 Division of Pulmonary and Critical Care Medicine, Department of Medicine, Mayo Clinic, Jacksonville, FL, United States; 5 Department of Pulmonary Medicine, Integrated Hospital Care Institute, Cleveland Clinic, Cleveland, OH, United States; 6 Division of Pulmonary, Critical Care and Sleep Medicine, University of South Florida, Tampa, FL, United States; 7 Division of Pulmonary, Critical Care and Sleep Medicine, Department of Medicine, University of Washington, Seattle, WA, United States; 8 Division of Pulmonary and Critical Care Medicine, Department of Medicine, University of Texas Health Sciences Center, San Antonio, TX, United States; 9 Department of Health Sciences Research, Mayo Clinic, Rochester, MN, United States; 10 Department of Transplantation, Mayo Clinic, Jacksonville, FL, United States; 11 Department of Cardiovascular Diseases, Mayo Clinic, Rochester, MN, United States; 12 Department of Cardiovascular Diseases, Mayo Clinic, Scottsdale, AZ, United States

**Keywords:** heart transplant, immunosupperssion, lung transplant, medication adherence, outcomes, transplant optimization

## Abstract

**Background:**

Medication nonadherence has been associated with poor outcomes in transplant recipients yet identifying these behaviors pre-transplant remains challenging. The simplified medication adherence questionnaire (SMAQ) is a brief, reliable instrument to assess medication adherence. The aim of this study was to perform SMAQ testing on thoracic organ candidates and examine correlations between these scores and individual patient factors predicting medication nonadherence.

**Methods:**

Consenting waitlisted lung, heart, and heart-lung transplant candidates from six thoracic transplant centers were mailed a survey that included demographic information and the Simplified Medication Adherence Questionnaire (SMAQ). Univariate logistic regression analysis was performed to assess association between pre-transplant medication adherence and demographic characteristics, psychosocial factors, and mortality.

**Results:**

Among 298 participants, 60.7% were medication nonadherent. Nonadherence was associated with some college education (OR: 1.73; CI: 1.06, 2.81), lower positive affect (OR: 0.95; CI: 0.92, 0.99), and higher uncertainty (OR: 1.02; CI: 1.01, 1.04). Higher positivity ratio predicted adherence.

**Conclusion:**

Identifiable patient-specific characteristics associated with medication nonadherence can be screened for during thoracic transplant evaluation. Interventions aimed at improving these factors during the pre-transplant period may improve clinical post-transplant outcomes, however, larger studies are needed to validate these findings.

## Introduction

Since the early days of thoracic transplantation, significant strides have been made in improving post-transplant survival of organ recipients, with the most recently available data revealing a median survival of 12.5 and 6.2 years for heart and lung transplant recipients, respectively (Khush et al., 2019; Chambers et al., 2021). While many individual factors have contributed to this marked improvement in outcomes, they are largely contingent on assumed sustained adherence to complex medication regimens that include immunosuppressive agents, antimicrobials, and other adjunct therapies. Indeed, poorer clinical outcomes have been repeatedly observed in medication non-adherent (MNA) thoracic transplant recipients, highlighting the importance of a robust understanding of factors associated with this phenomenon (Korb-Savoldelli et al., 2010).

While many centers view MNA as a relative contraindication to transplant, a major challenge for clinicians is to identify candidates at risk prior to surgery. Factors such as demographic information, socioeconomic status, and affect have been studied with various degrees of accuracy in predicting MNA in solid organ recipients (Colmenero et al., 2024; Corr et al., 2025; Denhaerynck et al., 2018; Bertram et al., 2019). Results of pre-transplant psychosocial assessments have also been shown to be predictive of post-transplant adherence (Dobbels et al., 2009). This link between underlying psychiatric disorders (such as anxiety and depression) and MNA has been well studied and for this reason routine pre-transplant screening is becoming more widely adopted (Rosenberger et al., 2016; Dew et al., 2015).

While post-transplant medication adherence has been relatively well studied, pre-transplant adherence remains less well characterized. This is particularly true amongst thoracic transplant candidates, although some available investigations have included this patient cohort. The Swiss Transplant Cohort Study studied both pre- and post-transplant adherence amongst renal, liver, lung, and heart recipients (de Geest et al., 2014) and found that pre-transplant MNA was a strong predictor of similar behavior in the post-transplant setting. An additional study including heart, lung, and liver transplant candidates described associations between education levels, amount of received practical and informational support, and personality trait conscientiousness on rates of pre-transplant MNA (Dobbels et al., 2005). Nöhre et al. (2021) utilized the Transplant Evaluation Rating Scale (TERS) in lung transplant candidates and found that the presence of pre-transplant psychosocial risk factors were associated with both lower rates of listing for surgery and higher levels of psychological outcomes such as depression and anxiety.

Given the complex landscape of risk-factors associated with MNA, clinicians need brief, practical tools that can identify patients at risk prior to transplant so that appropriate counseling and assistance can be provided. The simplified medication adherence questionnaire (SMAQ) has proven to be a short, reliable instrument designed to assess medication adherence and has been validated across a variety of medical conditions (Knobel et al., 2002; Ortega Suárez et al., 2011). Because pre-transplant thoracic transplant candidates are prescribed diverse medication regimens tailored to individual underlying disease states, we intentionally designed this study to capture global medication adherence trends across often heterogenous medication classes. The SMAQ is uniquely suited to address this broad question, whereas other post-transplant adherence tools typically focus on adherence to immunosuppressive agents in the post-transplant setting.

The primary aim of this study was to assess rates of medication adherence in thoracic transplant candidates using the SMAQ. Our secondary aim was to identify demographic, psychosocial, and clinical factors associated with MNA. Additionally, we examined whether pre-transplant adherence status was associated with key waitlist outcomes including organ transplantation, waitlist removal, or death. Based on existing findings in the literature, we hypothesized that greater illness uncertainty, higher negative affect scores, and symptoms consistent with anxiety or depression would be associated with higher rates of MNA.

## Materials and methods

The Mayo Clinic Institutional Review Board (IRB) approved this study under IRB 15-005378; individual sites obtained approval from their respective IRBs. This study adheres to the ethical standard of the Declaration of Helsinki.

### Study population

Eligible participants included consenting adult lung, combined heart-lung, and heart transplant candidates on the waiting list at Mayo Clinic Rochester, Mayo Clinic Arizona (heart only), or Mayo Clinic Florida. Lung transplant candidates on the waiting lists at University of Florida Gainesville; Cleveland Clinic Foundation; University of Washington; and University of Texas Health Science Center San Antonio who consented to sharing their contact information with Mayo Clinic for the purpose of research participation were also eligible. Patients were excluded if they required a medical interpreter or did not have a mailing address within the United States due to survey administration limitations. Recruitment occurred from September 16, 2015 to September 30, 2019.

### Questionnaire administration

Eligible participants were mailed a questionnaire that included the SMAQ with a cover letter describing that participation in the study was voluntary (patients could decline by not returning the survey) and a small token of appreciation (such as a parking voucher, cafeteria credit, or gift shop pass valued at approximately $10, depending on center availability). A second questionnaire was mailed to non-responders after 1 month if not transplanted or delisted/deceased. One month after the second mailing, continued non-responders were contacted by phone if not transplanted or delisted/deceased and offered the option to complete the questionnaire by phone or receive a third mailing. Responses to questionnaires were entered into a secured, web-based Research Electronic Data Capture (REDCap) database (Harris et al., 2009) hosted by Mayo Clinic.

### Data collection

Baseline patient characteristics including demographic variables, primary indication for transplant evaluation, and body mass index (BMI) were abstracted from the electronic medical record. Psychosocial Assessment of Candidate for Transplantation (PACT) and Stanford Integrated Psychosocial Assessment for Transplant (SIPAT) scores were likewise obtained from the electronic medical record. These assessments were completed by the transplant social work and/or transplant psychiatrist during routine clinical pre-transplant evaluations, independent of this research study. Both instruments were included in the analysis due to the variation in instrument use across participating centers. Follow-up data was collected through May 1, 2020. Mayo Clinic data was obtained from the electronic medical record. Other participating institutions provided the date of transplant listing, transplant surgery, delisting, and death, when applicable. Questionnaires included demographic information, relationship of the primary caregiver, and several psychometric survey instruments described below.

### Survey instruments

We selected the SMAQ to capture overall medication adherence across the diverse pre-transplant medication regimens often encountered in thoracic transplant candidates, rather than focusing on adherence to a specific drug class such as immunosuppressants. While transplant-specific adherence instruments exist (BAASIS, ITAS, etc.), we chose the SMAQ because it is practical for mailed surveys, has robust psychometric properties with validated diagnostic testing metrics, and because it captures both intentional and unintentional non-adherence. Given the established psychometric properties of the selected instruments across diverse populations, Cronbach’s alpha values reported for each instrument are derived from the published validation studies rather than calculated within the present cohort.

#### SMAQ

Medication adherence was assessed with the SMAQ, a previously validated six-item questionnaire (Cronbach’s α 0.75). Respondents answer four questions with yes/no answers addressing medication adherence and two questions regarding the number of times medications have been missed. Scoring of the questionnaire is binary; the respondent is either medication adherent or non-adherent. The SMAQ has a sensitivity of 72% and specificity of 91% for identifying medication non-adherence (Knobel et al., 2002).

#### PANAS

The Positive and Negative Affect Schedule (PANAS) was used to assess affect. PANAS is a previously validated 20-item scale (Cronbach’s α 0.86 positive affect, 0.84 negative affect). Respondents rate ten positive and ten negative words on a one to five Likert scale with one indicating that the word applies minimally to the respondent and five indicating that the word applies strongly to the respondent. Higher scores for positive words and negative words indicate more positive and negative affect, respectively (Crawford and Henry, 2004). Based on our prior research the minimal clinically important difference is 3.8 for positive affect scores and 3.0 for negative affect scores (Pennington et al., 2020). The positive to negative affect ratio is termed the positivity ratio (Bradburn, 1969; Fredrickson and Losada, 2005). A positivity ratio of ≥ 2.9 distinguishes individuals with greater resilience, better optimal functioning, and more social resources compared to their counterparts (Fredrickson and Losada, 2005).

#### GAD-2

We used the two-item, validated Generalized Anxiety Disorder Scale-2 (GAD-2) to screen for symptoms of anxiety (Cronbach’s α 0.82) (Skapinakis, 2007). A score of three or higher is a positive screen for anxiety with a sensitivity of 84.4% and a specificity of 72.8% for generalized anxiety disorder (Seo and Park, 2015).

#### PHQ-2

We used the validated, two-item Patient Health Questionnaire-2 (PHQ-2) to screen for depression (Cronbach’s α 0.88) (Arroll et al., 2010). A score of 3 or higher is considered a positive screen for depression with a sensitivity and specificity of 85.7% and 69.2% for major depressive disorder, respectively (Wang et al., 2015).

#### MUIS

Uncertainty was measured using the Mishel Uncertainty in Illness Scale (MUIS). MUIS is a 33-item scale with responses on a 1–5 Likert scale. Questions are divided into four-factor structure that includes ambiguity, complexity, inconsistency, and unpredictability (Cronbach’s α 0.87) (Mishel, 1981). Scores in each of the four factors are summed with a higher score indicating greater uncertainty.

### Data analysis

The primary outcome was to assess rates of medication adherence in thoracic transplant candidates using the SMAQ. Additionally, we evaluated possible factors associated with MNA including demographics, emotional health (PANAS, GAD-2, PHQ-2), and illness uncertainty (MUIS). Results are primarily presented as unit odds ratio (OR) with 95% confidence interval. Odds ratios were calculated with 95% confidence interval to determine the association of medication adherence with dichotomous variables. A two-sided *p*-value < 0.05 was considered statistically significant for all analyses. Secondary outcomes included removal from the waitlist, transplantation, and death. Binary logistic regression analysis was performed to determine the association of MNA with continuous variables. Multivariable modeling was not performed to avoid overfitting and to preserve statistical power for individual associations. JMP statistical software (version 9.01, SAS, Cary, NC) or SAS version 9.4 (SAS Institute Inc.; Cary, NC, USA) was used for data analysis.

### Missing data

Participants were only included in the analysis if a pre-transplant SMAQ was completed in its entirety. In the event that non-SMAQ data was missing, the participant was excluded from the corresponding analysis.

## Results

### Patient demographics

During the study period, 298 of the 327 (91.1%) patients approached completed the SMAQ and associated questionnaire. The cohort comprised 144 lung (48.3%), 148 heart (49.7%), and 6 combined heart-lung (2.0%) transplant candidates (Table 1). The median age at study enrollment was 61.2 years [interquartile range (IQR) 56.5, 65.9]. Most participants were male (63.4%), married (72.2%), White race (89.9%), and completed at least some college (65.4%). Spouses served as the primary caregiver (67.8%) for the majority of participants.

**TABLE 1:** Cohort characteristics.

	Median (IQR) or *n* (%)
*n*	298
Lung transplant candidates	144 (48.3)
Heart transplant candidates	148 (49.7)
Heart-lung transplant candidates	6 (2.0)
Age (yrs)	61.2 (56.5, 65.9)
Gender	
Male	189 (63.4)
Marital status	
Married	215 (72.2)
Education	
Less than some college	103 (34.6)
Some college or higher	195 (65.4)
Race	
Caucasian	268 (89.9)
Primary caregiver	
Spouse	202 (67.8)
CLung transplant candidates	
*n*	144
Reason for transplant	64*
IPF	26 (40.6)
COPD	21 (32.8)
Non-IPF ILD	11 (17.2)
Bronchiectasis	6 (9.4)
Unknown	80
Received transplant	93 (64.6)
Removed from waitlist	25 (17.4)
Died	27 (18.8)
CHeart transplant candidates	
*n*	148
Reason for transplant	
Dilated cardiomyopathy	69 (46.6)
Ischemic cardiomyopathy	40 (27.0)
Congenital heart disease	12 (8.2)
Hypertrophic cardiomyopathy	8 (5.4)
Other	19 (12.8)
Received transplant	79 (53.4)
Removed from waitlist	22 (14.9)
Died	22 (14.9)
CHeart-lung transplant candidates	
*n*	6
Reason for transplant	
Congenital heart disease	3 (50.0)
Primary pulmonary hypertension	2 (33.3)
Other	1 (16.7)
Received transplant	3 (50.0)
Removed from waitlist	1 (16.7)
Died	1 (16.7)

IQR, interquartile range; YRS, years; COPD, chronic obstructive pulmonary disease; ILD, interstitial lung disease; IPF, idiopathic pulmonary fibrosis.

*Reason for transplant unavailable for 80 lung transplant candidates.

Body mass index category varied by thoracic transplant type (*p* < 0.0001). The majority of heart transplant candidates were categorized as overweight (39.2%) or obese (42.6%) while the majority of lung transplant candidates were categorized as normal (36.1%) or overweight (45.8%).

The primary diagnosis prompting transplant evaluation was unavailable for lung transplant candidates enrolled in the study from non-Mayo sites (data unavailable for 80 of the 144 lung transplant participants). Idiopathic pulmonary fibrosis (IPF) (40.6%), chronic obstructive pulmonary disease/emphysema (32.8%), non-IPF interstitial lung disease (17.2%), and bronchiectasis (9.4%) were the primary diagnoses for the remaining 64 lung transplant candidates. The primary diagnosis for heart transplant candidates included dilated cardiomyopathy (46.6%), ischemic cardiomyopathy (27.0%), congenital heart disease (8.2%), and hypertrophic cardiomyopathy (5.4%). The majority of the heart-lung transplant candidates were listed for transplant secondary to congenital heart disease (50.0%) or primary pulmonary hypertension (33.3%).

As of May 1, 2020, 175 (58.7%) participants were transplanted (93 of 144 lung, 79 of 148 heart, and 3 of 6 combined heart-lung) and 48 (16.1%) participants were removed from the transplant list (25 lung, 22 heart, and 1 combined heart-lung) (Table 1). At the end of the study period, 50 (16.8%) participants had died (27 lung, 22 heart, and 1 combined heart-lung). The majority of deaths occurred prior to transplantation (29 of 50 deaths). Only 15 deaths were reported within the first-year post-transplant (1-year post-transplant mortality rate: 8.6%).

### Medication non-adherence and candidate characteristics

The majority of thoracic transplant candidates (60.7%) were medication non-adherent (Table 2). No difference in medication adherence between heart transplant (98 of 144, 66.3% medication non-adherent) and lung transplant (81 of 148, 56.3% medication non-adherent) candidates was observed (OR: 1.52; CI: 0.95, 2.45). Candidates that received at least some college education were more likely to be medication non-adherent (127 of 195, 65.1%) compared to candidates without any college education (53 of 102 participants, 52.0%; OR: 1.73; CI: 1.06, 2.81). There was no difference in medication adherence according to age, sex, marital status, race, or BMI. Likewise, pre-transplant psychosocial assessment with the PACT (OR: 0.93; CI: 0.59, 1.46) and SIPAT (OR: 1.01; CI: 0.97, 1.05) were not associated with MNA (Tables 3, 4).

**TABLE 2:** Nominal candidate characteristics and odds of medication nonadherence.

Factor	OR (CI)
Thoracic transplant type^+^	1.52 (0.95, 2.45)
At least some college education	1.73 (1.06, 2.81)*
Male sex	1.53 (0.94, 2.49)
Married	1.18 (0.71, 1.98)
Race (Caucasian compared to non-Caucasian)	0.75 (0.33, 1.67)
Overweight or obese	1.58 (0.95, 2.64)
Positivity ratio ≥ 2.9	0.46 (0.28, 0.77)
GAD-2 score ≥ 3	1.22 (0.64, 2.31)
PHQ-2 score ≥ 3	1.71 (0.79, 3.72)

CI, confidence interval; GAD-2, Generalized Anxiety Disorder Scale-2; OR, odds ratio; PHQ-2, Patient Health Questionnaire-2.

*Denotes statistical significance. ^+^Heart transplant candidates compared to lung transplant candidates.

**TABLE 3:** Continuous variables and odds of medication nonadherence by univariate logistic regression.

Factor	Unit OR (CI)
Age	0.98 (0.96, 1.00)
BMI	1.01 (0.96, 1.06)
PACT score	0.93 (0.59, 1.46)
SIPAT score	1.01 (0.97, 1.05)
Positive affect score	0.95 (0.92, 0.99)*
Negative affect score	1.05 (1.01, 1.10)*
Positivity ratio	0.67 (0.53, 0.85)*
GAD-2 score	1.23 (1.04, 1.04)*
PHQ-2 score	1.20 (0.98, 1.46)
MUIS score	1.02 (1.01, 1.04)*
MUIS complexity score	1.10 (1.03, 1.17)*
MUIS ambiguity score	1.02 (0.99, 1.05)
MUIS unpredictability score	1.06 (1.00, 1.14)
MUIS inconsistency score	1.05 (0.99, 1.11)

BMI, body mass index; CI, confidence interval; GAD-2, Generalized Anxiety Disorder Scale-2; MUIS, Mischel Uncertainty in Illness Scale; OR, odds ratio; PACT, Psychosocial Assessment of Candidate for Transplantation; PHQ-2, Patient Health Questionnaire-2; SIPAT, Stanford Integrated Psychosocial Assessment for Transplant.

*Denotes statistical significance.

**TABLE 4:** Questionnaire scores by medication adherence status.

	Mean (SD)	
*n*	Adherent (181)	Nonadherent (117)
PACT score	2.43 (0.60)	2.43 (0.72)
SIPAT score	14.22 (7.56)	13.10 (8.35)
Positive affect score	37.50 (6.94)	34.86 (8.14)
Negative affect score	16.36 (5.96)	18.29 (6.42)
GAD-2 score	0.99 (1.38)	1.38 (1.60)
PHQ-2 score	0.87 (1.27)	1.13 (1.34)
MUIS score	74.17 (15.75)	78.84 (14.39)
MUIS complexity score	12.65 (3.94)	14.19 (3.98)
MUIS ambiguity score	33.40 (8.92)	34.94 (8.00)
MUIS unpredictability score	15.81 (3.95)	16.62 (3.25)
MUIS inconsistency score	12.16 (4.43)	13.09 (3.88)

GAD-2, Generalized Anxiety Disorder Scale-2; MUIS, Mischel Uncertainty in Illness Scale; OR, odds ratio; PACT, Psychosocial Assessment of Candidate for Transplantation; PHQ-2, Patient Health Questionnaire-2; SD, standard deviation; SIPAT, Stanford Integrated Psychosocial Assessment for Transplant.

### Medication non-adherence and emotional health

Thoracic transplant candidates with lower positive affect scores (OR: 0.95; CI: 0.92, 0.99), and conversely those with higher negative affect scores (OR: 1.05; CI: 1.01, 1.10) were more likely to be medication non-adherent. Those with lower positivity ratio (OR: 0.67; CI: 0.53, 0.85) were more likely to be medication non-adherent. Candidates who met the critical threshold of positivity ratio ≥ 2.9 were significantly less likely to be medication non-adherent (OR: 0.46; CI: 0.28, 0.77).

The odds of MNA increased with GAD-2 scores (OR: 1.23; CI: 1.04, 1.48) but was not associated with PHQ-2 scores (OR: 1.20; CI: 0.98, 1.46). A positive GAD-2 (OR: 1.22; CI: 0.64, 2.31) or PHQ-2 (OR: 1.71: CI: 0.79, 3.72) test, using the previously validated cutoff of ≥3, was not associated with MNA (Seo and Park, 2015; Wang et al., 2015).

The odds of medication non-adherence increased with total MUIS score (OR: 1.02; CI: 1.01, 1.04) and the MUIS sub-score of complexity (OR: 1.10; CI: 1.03, 1.17). MUIS sub-scores of ambiguity (OR: 1.02; CI: 0.99, 1.05), unpredictability (OR: 1.06; CI: 1.00, 1.14), and inconsistency (OR: 1.05; CI: 0.99, 1.11) were not associated with medication non-adherence.

### Outcome measures and medication non-adherence

Medication non-adherent transplant candidates (98 of 181, 54.1%) were less likely to receive a transplant during the study period compared to medication adherent candidates (77 of 117, 65.8%; OR: 0.61; CI: 0.38, 0.99) (Table 5). There was no difference in removal from the waitlist (non-adherent 32 of 181, 17.7%; adherent 16 of 117, 13.6%; OR: 1.36; CI 0.71, 2.60), overall death (non-adherent 27 of 181, 14.9%; adherent 23 of 117, 19.7%; OR: 0.72; CI: 0.39, 1.32), and 1-year post-transplant death (non-adherent 5 of 97, 5.2%; adherent 10 of 77, 13.0%; OR: 0.36; CI: 0.12, 1.11) between medication non-adherent and medication adherent transplant candidates.

**TABLE 5:** Medication nonadherence and odds of outcome of interest.

Outcome	OR (CI)
Organ transplant	0.61 (0.38, 0.99)*
Removal from waitlist	1.36 (0.71, 2.60)
Death	0.72 (0.39, 1.32)
1-year post-transplant death	0.36 (0.12, 1.11)

CI, confidence interval; OR, odds ratio.

*Denotes statistical significance.

## Discussion

Our findings suggest that medication non-adherence is common among thoracic transplant candidates and no different between heart or lung transplant candidates, with over 60% identified as non-adherent using the SMAQ in this cohort. This is consistent with the existing literature on patients that have multimorbid disease and other cohorts of solid organ transplant recipients (Foley et al., 2021; Maksyutynska et al., 2025; Springfield-Trice et al., 2025). Patients with a high positivity affect ratio were more likely to be adherent to medications. Some college education, higher levels of uncertainty (particularly in the complexity dimension), higher negative affect score, and lower positivity ratio were associated with decreased rates of medication adherence. Demographic variables such as age, sex, race, marital status, and BMI did not have a significant impact on medication adherence. Notably, frequently utilized pre-transplant psychiatric assessments such as the PACT and SIPAT scores did not correlate with rates of medication adherence. Overall, candidates that had higher rates of MNA were less likely to receive an organ transplant, although there were no significant differences in waitlist removal, death, or 1-year mortality.

Notably, positive screens on GAD-2 and PHQ-2 were not significantly associated with adherence when treated as binary variables, despite continuous GAD-2 scores showing association. One possible explanation for this is that by their nature they are brief screening tools and lack the diagnostic sophistication of their complete scale counterparts (GAD-7 and PHQ-9). Unfortunately, the results of these more in-depth assessments are unavailable for us to evaluate.

It is unclear exactly why those with some college education are more likely to be non-adherent to medical therapy. This initially seems paradoxical, but similar findings have been observed elsewhere both in pretransplant populations and amongst other medical conditions (Tomar et al., 2019; Dobbels et al., 2005). One hypothesis is that those with a higher level of education may be more likely to question their healthcare teams and form their own second opinion that is contradictory, while those with a lower level of education may be more trusting of those same recommendations. Another possibility is that more highly educated individuals may have more competing demands and time constraints that may lead to unintentional non-adherence. These hypotheses remain speculative and cannot be confirmed from our data alone. Further research should be performed to validate this association and determine if interventions targeted to this group of patients have an impact on overall rates of adherence.

Our study is unique in that it represents one of the largest cohorts of its type in the thoracic transplant literature and to our knowledge is the first to assess the performance of SMAQ in this population. It also utilizes a broad variety of surveys in an attempt to analyze many of the known unique contributors to MNA. As shown by the lower rates of organ transplant in patients with lower rates of medication adherence, clinicians view MNA as a significant risk factor that could lead to complication or graft loss. By targeting our intervention to patients that have not yet undergone transplant, we aimed to inform potential evidence-based practices to improve post-transplant outcomes through early identification and support of patients at-risk for developing MNA. This is supported by evidence available in the literature which shows that pre-transplant adherence behaviors are predictive of post-transplant adherence (Dobbels et al., 2009; de Geest et al., 2014). Our hope is that identifying psychosocial characteristics associated with MNA will enable transplant teams to better recognize candidates who may benefit from targeted behavioral or psychosocial interventions designed to enhance adherence and optimize transplant readiness.

### Strengths

Our article has several strengths. First, we observed an excellent response rate, with 91.1% of candidates completing the questionnaires provided. Second, it utilizes a wide array of surveys and assessments to identify many possible factors related to rates of medication adherence. And finally, it represents a large cohort of patients across the United States which increases generalizability of our findings to other transplant centers.

### Limitations

Our study had limited post-transplant deaths during the study period and as such we were likely underpowered to demonstrate an association with post-transplant mortality. Our use of the SMAQ may limit direct comparisons with available studies that employed more transplant-specific medication adherence instruments. While the SMAQ provides a validated measure of global medication adherence, future studies comparing the SMAQ to transplant-specific adherence instruments may allow for more direct comparisons. Additionally, as the analyses were univariate, the findings should be interpreted within this context. Future studies with larger sample sizes should employ multivariable modeling to assess potential confounding among correlated predictors. Nonetheless, the results remain meaningful given the scarcity of existing data on this topic. In addition, our cohort was predominantly white and married which may limit the generalizability of our findings to other populations. Finally, although the sickest thoracic transplant candidates were not excluded, their participation was likely limited both due to decreased capacity to complete the surveys and increased transplant urgency resulting in shorter waiting times.

## Conclusion

This multicenter cohort study demonstrates that medication non-adherence is highly prevalent among thoracic transplant candidates and provides a broad assessment of patient-specific characteristics associated with MNA prior to thoracic organ transplantation. Treatment teams may use these findings to proactively screen for candidates displaying psychosocial characteristics linked to higher risk, such as lower positive affect or greater illness uncertainty, and to consider implementing individualized behavioral or psychosocial interventions to support adherence during the transplant evaluation process. Future research should seek to validate these findings and evaluate targeted interventions designed to modify these psychosocial factors, with the goal of enhancing adherence and promoting improved long-term outcomes following transplantation.

## Data Availability

The raw data supporting the conclusions of this article will be made available by the authors, without undue reservation.
